# Wild Animals and Their Tamers

**Published:** 1882-05

**Authors:** 


### WILD ANIMALS AND THEIR TAMERS.

"Gentle?" remarked one of these venturesome folk the other day. "Those tigers of mine? Why, do you see that whip? I know, as well as I know anything, that if I drop that whip when I am in that cage, they'll be on me. Their idea of obedience is connected with the whip, first; then with my voice; then with my face. Severity? Cruelty? No use at all. I never u e cruelty in training them. Only patience. When I take on a new cage of beasts I work to get them used to me; feeding them; cleaning the cage; talking to them; all that sort of thing; before I go in among them. Then I do that. It's a ticklish piece of business, going in the first time; and I pick my chance for it when they're specially peaceable. I go right in, just as if it were a matter of course, but I keep my eyes about me. It's all humbug that a man's eye has any power over a wild beast. Your eyes are to watch their motions—that's all. They'll find out quickly enough if you're getting careless. They're sure enough to be watching you all the time. Are they intelligent? Well, there's as much difference among 'em as there is among men. I can train a really intelligent lion, right from the wild, in about four weeks, so he will do all that the lion kings make them do. A lioness always takes a couple of weeks longer, and so does a leopard or a tiger. You can't get a hyena well in hand inside of two months. They're the meanest of brutes. They never understand anything but a club. The easiest to train, because they know the most, are pumas. I can teach a puma all it needs to know in four weeks. Affection? Teach those fellows to love you? That's all nonsense. They'll fawn and fawn on you, and you'll think you've done it, maybe. Then you go into the cage, if you want to, without your whip or when they're in bad temper, and find out for yourself what they'll do. See that dent in the side of my head and those deep scars on my arm! There are more down here"—patting his leg. "Got 'em from the best-trained lions you ever saw. It's awful, sometimes, to have one of those fellows kind o' smell of you and yawn, and shut his jaws, say, close to one of your knees! See my wife there? She's the 'Panther Queen,' just as I'm a 'Tiger King,' and that fellow yonder's a 'Lion King.' Her pets are playing with her now, but they've scratched her well, I tell you. There's great odds among them, though, and that young puma with her head up to be kissed is what you might call gentle. Only they're all treacherous. Every lion king gets sick of it after awhile. I could name more than a dozen of the best, who have given it up right in the prime of life. Once they give it up, nothing'll tempt 'em inside of a cage again. You see, every now and then, some other tamer gets badly clawed and bitten. They've all been clawed and bitten more or less themselves. The strain on a man's nerves is pretty sharp—sure death around him all the while. And the pay isn't anything like what it was.—*St. Nicholas.*

